# Complicated mycotic saccular aneurysm of the infra-renal abdominal aorta with infected retroperitoneal hematoma: a clinical case report

**DOI:** 10.3389/fcvm.2024.1497561

**Published:** 2024-11-21

**Authors:** A. M. Chinaliyev, Y. I. Jussubaliyev, D. T. Khassenov, D. Sabyruly, E. M. Shaimerden, M. S. Kapyshev, R. I. Aleushinov, S. Sh. Aitkul, G. A. Yessenbayeva, I. N. Sagandykov, T. A. Sultanaliyev

**Affiliations:** National Research Oncology Center, Astana, Kazakhstan

**Keywords:** abdominal aortic aneurysm, aneurysm rupture, retroperitoneal hematoma, infection, bypass surgery, hybrid surgical procedures

## Abstract

**Background:**

Rupture of an abdominal aortic aneurysm (AAA) is a life-threatening emergency, with untreated cases nearing a 100% mortality rate. This case presents a rare complication of AAA rupture with an infected retroperitoneal hematoma, emphasizing the importance of timely diagnosis and a multidisciplinary approach.

**Case presentation:**

A 59-year-old male presented with lower back pain, fever, and difficulty moving, persisting for three weeks. Imaging revealed a saccular infra-renal AAA rupture with an infected retroperitoneal hematoma. Emergency surgery included extracorporeal subclavian-femoral bypass and aneurysm resection. Despite intraoperative complications, the patient recovered after 16 days in intensive care and was discharged in satisfactory condition. Follow-up CT one month later showed functioning bypasses and clinical improvement.

**Conclusion:**

This case illustrates the critical need for early diagnosis and coordinated surgical intervention in complex AAA ruptures complicated by infection. Timely multidisciplinary treatment is crucial to prevent further complications and improve patient outcomes.

## Introduction

Abdominal aortic aneurysm (AAA) rupture is a life-threatening emergency that requires immediate intervention, with untreated cases carrying a mortality rate of up to 90%. While most AAA ruptures involve the posterior aortic wall and cause retroperitoneal hematomas, which may temporarily limit bleeding, the presence of an infected retroperitoneal hematoma introduces significant complications, including a heightened risk of sepsis and increased complexity in surgical management. This case report presents the rare scenario of a ruptured infra-renal AAA complicated by both an infected retroperitoneal hematoma and a history of lumbar spinal tuberculosis.

AAAs are typically asymptomatic until rupture and occur more frequently in men between 65 and 85 years old. Risk factors such as atherosclerosis, hypertension, and smoking play a critical role in aneurysm formation, with a critical rupture risk once the aneurysm exceeds 5.5 cm. However, this case underscores the need to consider non-atherosclerotic factors such as infections in aneurysm development. Lumbar spinal tuberculosis, in this case, may have weakened the aortic wall, contributing to aneurysm formation and eventual rupture.

Open surgery remains the gold standard for ruptured AAA repair, especially in the presence of infection, where vascular bypass techniques like subclavian-femoral bypass are employed to bypass the infected area. The patient's postoperative recovery and the multidisciplinary approach highlight the importance of early diagnosis and prompt surgical intervention in managing complex AAA cases, especially those with complicating factors such as infection or atypical presentations. This case provides valuable insights for clinicians managing similarly complex presentations.

## Clinical case presentation

Patient E, a 59-year-old male, was admitted to the Center for Vascular Surgery at the National Scientific Oncology Center in critical condition.

*Chief complaints on admission*: The patient reported lower back pain, difficulty with movement, and a persistent fever of 38°C lasting for the past three weeks.

*Medical history* included Type 2 Diabetes Mellitus in a state of subcompensation, complicated by diabetic polyneuropathy of the lower extremities, ischemic heart disease with post-myocardial infarction cardiosclerosis, and a history of coronary artery bypass grafting (CABG) in 2015. Additionally, he had grade 3 arterial hypertension with very high cardiovascular risk and chronic heart failure, New York Heart Association (NYHA) Class II. His psychosocial history was unremarkable, with no familial or genetic disorders reported. The patient had been a smoker for 20 years but had quit smoking 15 years ago, and there was no family history of aneurysms or other vascular diseases.

*History of Present Illness:* The patient reported that symptoms had been present for 23 days, which he attributed to physical exertion and possible heavy lifting at home. Initially, the patient was admitted to a local hospital for inpatient treatment but was later transferred to a specialized facility.

**Figure 1:**
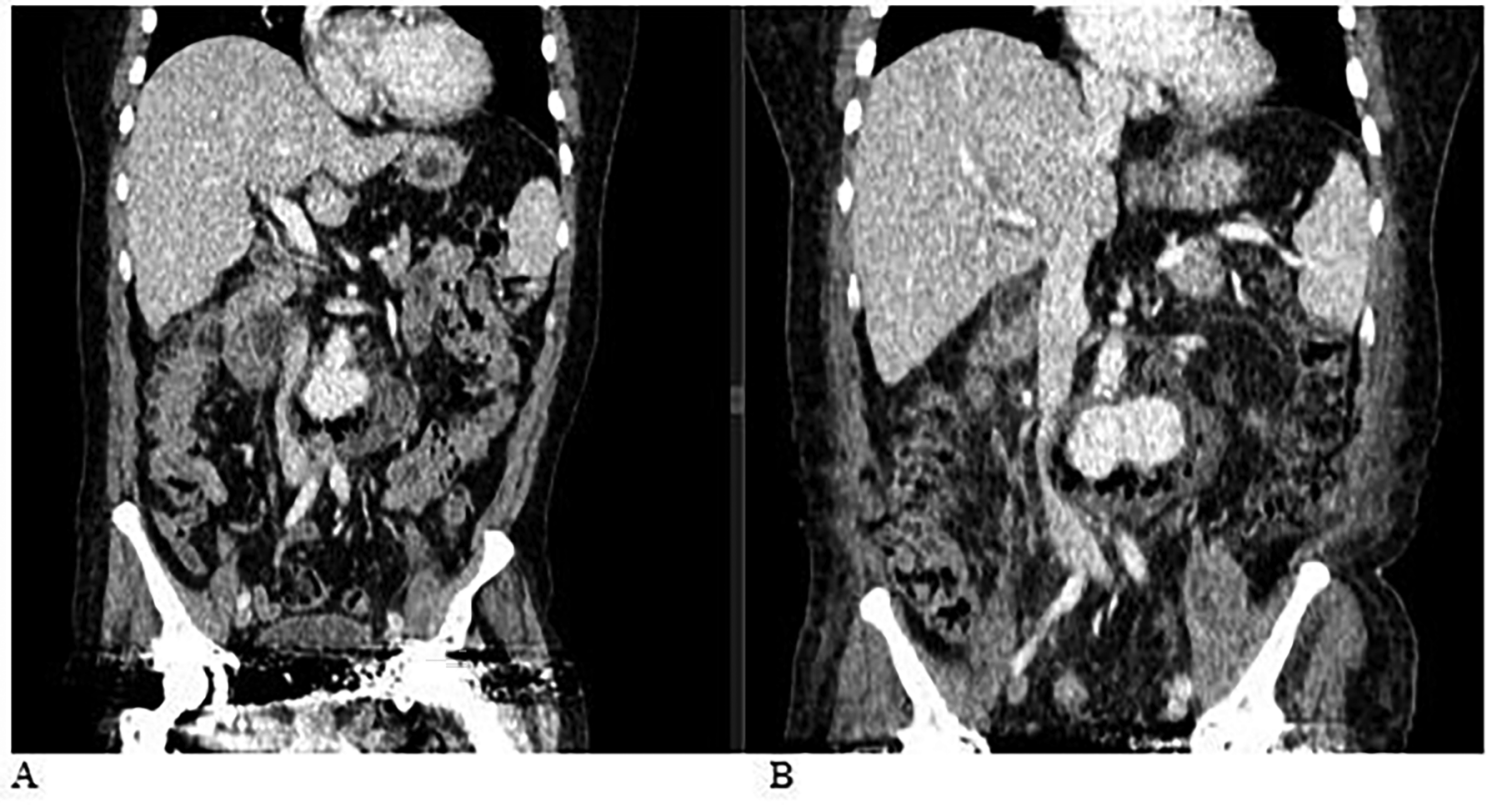
**(A)** Computed tomography (CT) scan of the abdominal organs and retroperitoneal space with contrast: A saccular aneurysm in the infra-renal part of the aorta along the posterior wall, measuring 3.4 × 2.9 × 2.7 cm. The width of the neck is 1.7 cm, and its extent is 2.0 cm. There is an area of infiltration around the described aneurysm, extending to the left psoas major muscle, with a length of approximately 13.0 cm. **(B)** Computed tomography (CT) scan of the abdominal organs and retroperitoneal space with contrast, performed dynamically after 4 days. Compared to the previous CT, there is a negative trend due to the increased size of the aneurysm and the abscess.

**Figure 2:**
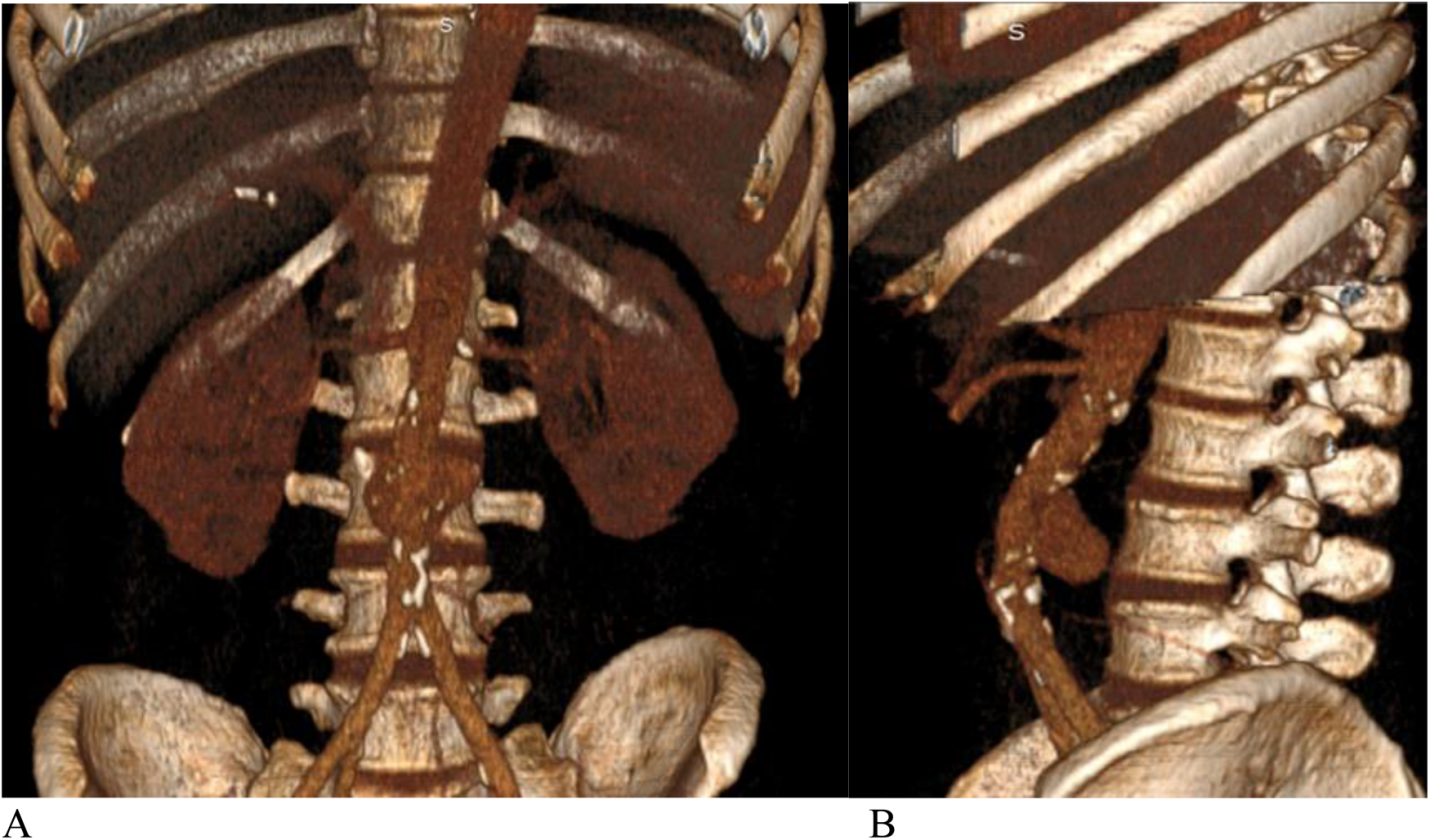
**(A)** Front view, **(B)** side view.—3D CT angiography. Saccular aneurysm of the infra-renal aorta with rupture of the posterior wall.

At the local hospital, a contrast-enhanced computed tomography (CT) scan of the abdominal organs and retroperitoneal space was performed (Figures 1, 2). The scan revealed a saccular aneurysm in the infra-renal part of the aorta, located along the posterior wall. The aneurysm measured 3.4 × 2.9 × 2.7 cm, with a neck width of 1.7 cm and an extent of 2.0 cm (Figures 1A, 2). Additionally, there was infiltration around the aneurysm, extending to the left psoas major muscle, measuring approximately 13.0 cm in length.

A multidisciplinary consultation was conducted, and the clinical diagnosis was confirmed as multifocal atherosclerosis with a saccular aneurysm of the infra-renal aorta, complicated by rupture into the retroperitoneal space, formation of a hematoma, and subsequent infection.

Based on the CT scan results (Figure 1B) of the abdominal organs and retroperitoneal space, which showed negative progression with an increase in the size of the aneurysm and associated abscess, along with worsening post-hemorrhagic anemia (hemoglobin levels dropping to 84–87 g/L), the risk of complete rupture of the infra-renal aortic aneurysm necessitated emergency surgery.

The patient was promptly taken for urgent surgical intervention, which included the following procedures:

• Extracorporeal subclavian-femoral bypass on the right side.
• Extracorporeal femoral-femoral bypass from right to left.
• Left lumbarotomy.
• Resection of the abdominal aortic aneurysm.
• Ligation of the infra-renal segment of the abdominal aorta.
• Opening and drainage of the retroperitoneal hematoma.

*Intraoperatively*: Under general anesthesia, the subclavian artery was exposed in the right supraclavicular area, and the site for an anastomosis was prepared. Clamps were applied to the subclavian artery, followed by a longitudinal arteriotomy. A proximal anastomosis with a 7 mm “Vascutek” allograft was created in an end-to-side configuration using 6/0 Prolene sutures, and the graft was tunneled subcutaneously to the right groin area. Simultaneously, in the upper thirds of both thighs, along the line of Scarpa's triangle, the common femoral, deep femoral, and superficial femoral arteries were exposed. Pulsation in the common femoral artery was satisfactory on both sides.

**Figure 3:**
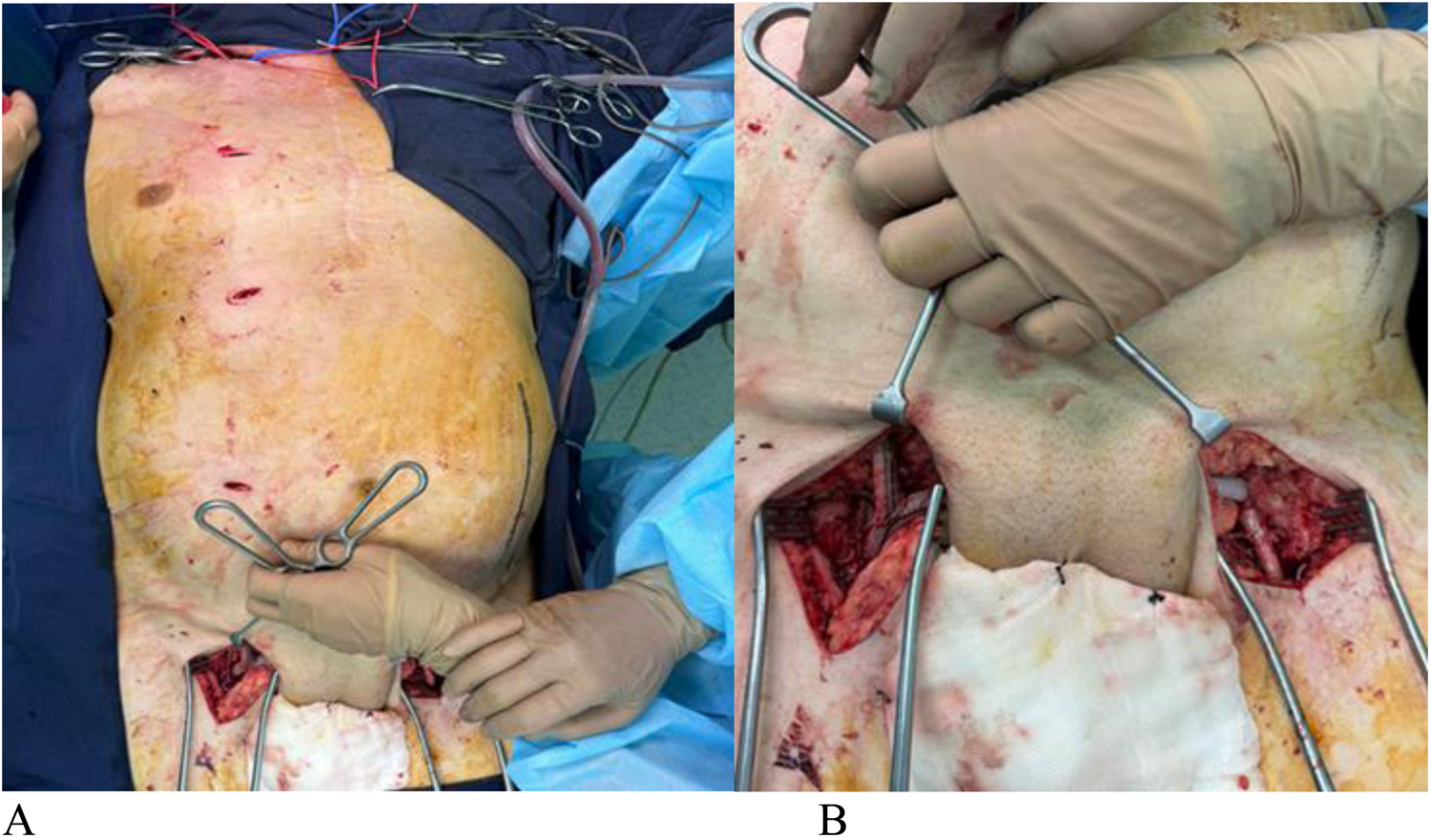
**(A,B)** sequence of the operation.

On the right side, clamps were applied to the common femoral, deep femoral, and superficial femoral arteries, and a proximal anastomosis with a 7 mm “Vascutek” allograft was created in an end-to-side configuration with the common femoral artery, using 6/0 Prolene sutures. Once the anastomosis was complete, clamps were removed from the right-side arteries (Figure 3A). The allograft was tunneled subcutaneously over the pubic bone. On the left side, clamps were similarly applied to the common femoral, deep femoral, and superficial femoral arteries. An arteriotomy was performed on the left common femoral artery above the origin of the deep femoral artery, and a distal anastomosis with an 8 mm “Vascutek” allograft was created, again using 6/0 Prolene sutures. After ensuring satisfactory blood flow and preventing air embolism, the clamps were removed, and satisfactory pulsation was observed below the anastomoses on both sides (Figure 3B).

A subclavian-prosthetic (femoral-femoral) anastomosis was then performed in an end-to-side configuration with 6/0 Prolene sutures, restoring main blood flow with air embolism prevention.

**Figure 4:**
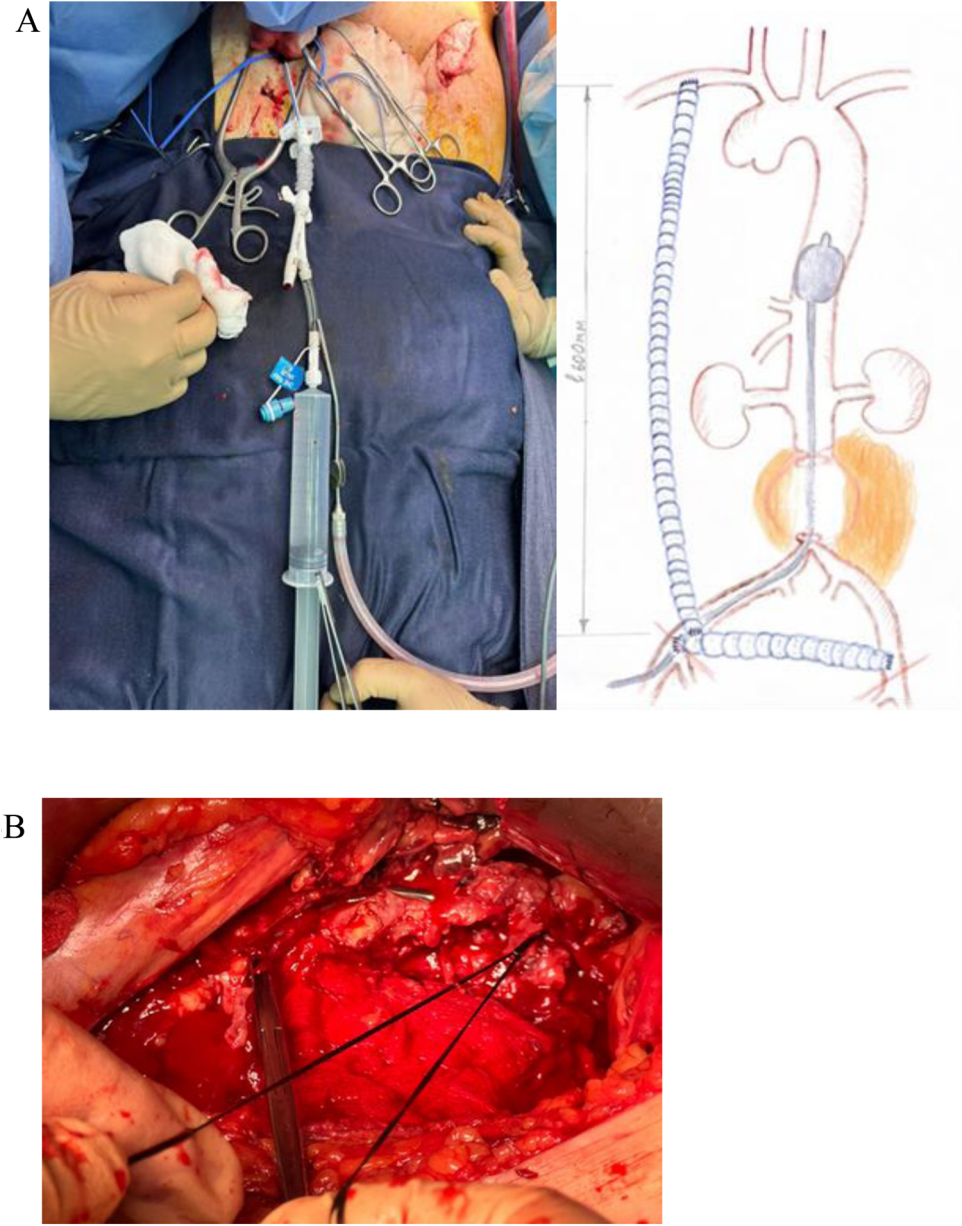
**(A)** the aortic balloon catheter was placed in the descending aorta above the rupture site through the right common femoral artery. **(B)** Ligation of the infra-renal segment of the abdominal aorta below the renal arteries.

Next, the abdominal aortic aneurysm was accessed via a pararectal approach. Despite technical difficulties and the presence of a surrounding hematoma, the aneurysm was exposed. To prevent massive blood loss, an aortic balloon catheter was inserted into the descending thoracic aorta through the right common femoral artery in collaboration with interventional radiologists. During the exposure of the aneurysm, massive bleeding occurred from the rupture site. The balloon catheter was positioned in the descending aorta above the rupture to control the hemorrhage (Figure 4A). The aortic walls were found to have atherosclerotic changes. The aneurysm neck was exposed below the renal arteries, and clamps were applied both above and below the aneurysm (Figure 4B).

Following the intravenous administration of 5,000 units of Heparin, thrombus masses were removed from the aneurysm cavity. The aortic walls were thin and fragile due to atheromatosis, which contributed to their tendency to tear. The infra-renal segment of the abdominal aorta was ligated below the renal arteries, effectively stopping the bleeding. Blood flow was restored, and pulsation below the anastomoses was confirmed as satisfactory. Hemostasis was achieved throughout the procedure.

The surgical wounds were closed in layers, and the retroperitoneal space and anastomoses were drained with silicone tubes. Povidone-iodine was applied, and the surgical sites were dressed in aseptic dressings. The estimated volume of blood loss during the surgery was 2,500 ml.

*Microbiological Sample Collection:* Intraoperatively, tissue samples were collected for microbiological and histological evaluation to ensure accurate pathogen identification:

• Wound Exudate Culture (Date: 30.10.23): *Escherichia coli* was detected at 10^6 CFU, with sensitivity to a broad spectrum of antibiotics including Ampicillin, Cefazolin, Gentamicin, Amikacin, and Meropenem, among others.
• Bronchial Lavage Fluid Culture (Date: 31.10.23): *Pseudomonas aeruginosa* was isolated at 10^3 CFU, with sensitivity to Ceftazidime, Gentamicin, Piperacillin/Tazobactam, and Ciprofloxacin, among other agents.
• Histological Analysis (Date: 31.10.23):
  ○ Macroscopic Findings: Thrombotic masses with associated vascular wall fragments (up to 5.5 × 3.0 × 0.6 cm), reddish in color.
  ○ Microscopic Findings: Thin fragments of the vascular wall showed signs of fibrosis, leukocyte debris, hemorrhage, and focal wall dissection. Thrombotic areas demonstrated dense fibrin networks intermixed with erythrocytes.
  ○ Conclusion: The morphology aligns with the diagnosis of infected aneurysmal tissue.

*Postoperative Care:* Following surgery, the patient was in the ICU for 16 days. His postoperative care included anticoagulant therapy, antibiotics (specifically cephalosporins and metronidazole), gastroprotective therapy (proton pump inhibitors), pain management, and daily wound dressings. Respiratory rehabilitation was also undertaken. During the early postoperative period, the patient experienced an increase in body temperature, managed with analgesics and antipyretics.

The patient developed bilateral lower lobe pneumonia and bilateral exudative pleuritis, contributing to respiratory failure, necessitating non-invasive mechanical ventilation. Acute renal failure (stage 2–3 KDIGO) was managed with intermittent hemodiafiltration; creatinine levels eventually normalized, and acute renal failure regressed. Post-hemorrhagic anemia persisted but was classified as mild and managed conservatively with blood transfusions and iron supplementation. The patient also experienced subfebrile fever, which lasted approximately one month but was managed with analgesics and antipyretics.

## Antibiotic therapy

• Preoperative Antibiotic Therapy (from 24.10.2023 to 28.10.2023): Piperacillin/Tazobactam 4.5 g in 250 ml of 0.9% sodium chloride, administered intravenously three times daily.
• Postoperative Antibiotic Therapy in ICU (from 29.10.2023 to 19.11.2023): Meropenem 2 g intravenously every 12 h over a 3-hour extended infusion, and Linezolid 600 mg intravenously every 12 h over 30–120 min.
• Postoperative Antibiotic Therapy in Ward (from 20.11.2023 to 27.11.2023): Piperacillin/Tazobactam 4.5 g in 250 ml of 0.9% sodium chloride, administered intravenously three times daily.

### Postoperative antithrombotic treatment

Post-surgery, a staged antithrombotic regimen was implemented:

• 28.11.2023–15.11.2023: Heparin at 20,000 IU in 4 ml of 0.9% sodium chloride solution, administered intravenously.
• 15.11.2023–20.11.2023: Enoxaparin (Clexane) 4000 anti-Xa IU (40 mg) subcutaneously, once daily.
• 20.11.2023–27.11.2023: Clopidogrel 75 mg, orally, once daily.

*Health indicators and laboratory data during hospitalization:* In the early postoperative period, the patient experienced an increase in body temperature, which was managed with analgesics and antipyretics. A subfebrile temperature persisted for about a month postoperatively. While in the intensive care unit, the patient exhibited instability in both blood pressure and heart rate, which later stabilized in the specialized department. Early postoperative signs of acute renal failure were noted but resolved after correction, with creatinine levels returning to normal. The general blood test (GBT) showed leukocytosis and elevated C-reactive protein (CRP) levels, indicative of intoxication syndrome. By the time of discharge, GBT, biochemical blood analysis, and CRP levels had returned to normal (see in Supplementary Material).

**Figure 5:**
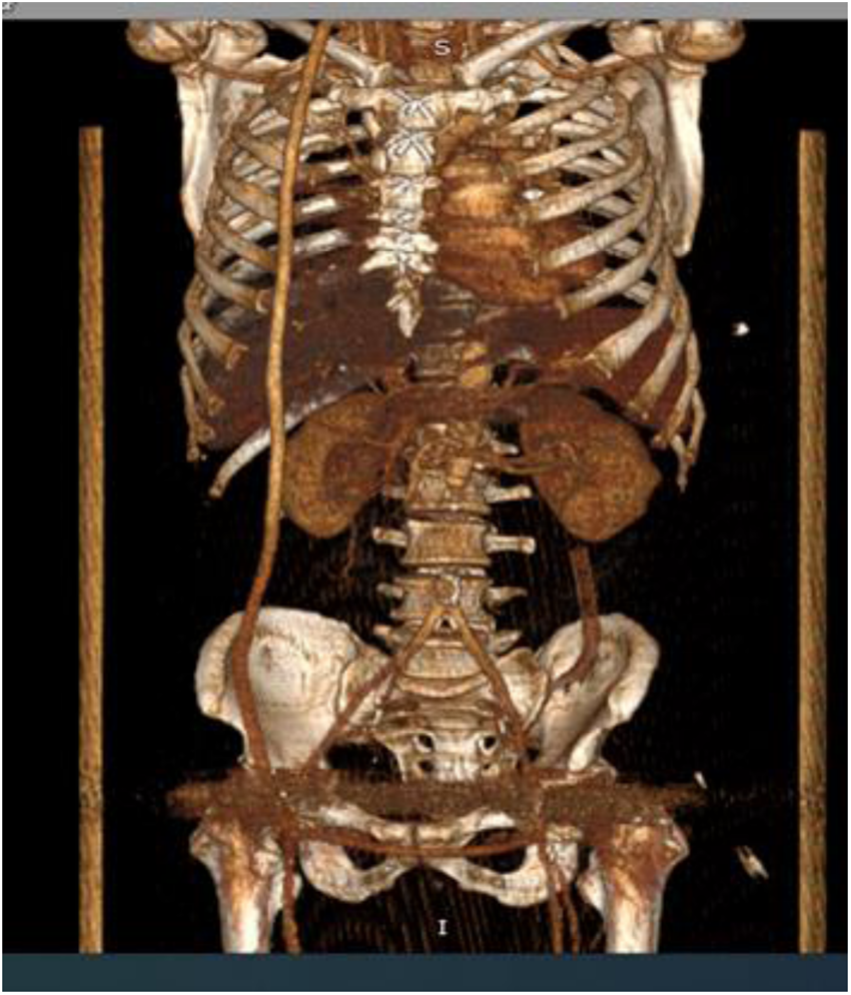
Ct with after one month. 3D reconstruction. The subclavin-femoral and femoral-femoral shunts are functioning.

*Follow-up and Outcomes:* One-month post-surgery, a follow-up CT scan (Figure 5) with contrast confirmed satisfactory function of all bypass grafts. The patient's hemoglobin and CRP levels normalized, and his physical activity levels increased. Despite residual weakness, he prepared to return to work while continuing to use a cane for mobility. Ten months later, he sought treatment for nonspecific spondylitis of the L3-L4 vertebrae, which was successfully managed. His overall health remained stable, with functional bypass grafts and no significant complaints. Long-term follow-up is crucial for managing his underlying conditions.

The patient adhered to prescribed therapeutic interventions, including medication and rehabilitation. Adherence was monitored through regular follow-ups and laboratory tests, confirming the effectiveness of treatments. The patient tolerated anticoagulant therapy without significant side effects, and no new complications arose during the recovery period. The patient's prognosis is considered favorable due to the successful surgical intervention and gradual recovery. Continued monitoring is necessary due to the complexity of his underlying conditions, including diabetes and ischemic heart disease.

## Discussion

This case presents a rare and challenging instance of an abdominal aortic mycotic aneurysm rupture complicated by an infected retroperitoneal hematoma. The patient's symptoms, including lower back pain, difficulty moving, and fever, alongside imaging findings of a saccular aneurysm measuring 3.4 × 2.9 × 2.7 cm and significant retroperitoneal infiltration, necessitated urgent surgical intervention.

Rupture of an abdominal aortic aneurysm (AAA) is an emergency situation, and if not recognized and treated promptly, it leads to significant morbidity and mortality, with untreated mortality approaching 100%. Most ruptures occur into the retroperitoneum, causing symptoms such as pain, dizziness, and a pulsating abdominal mass. AAAs most often occur in men aged 65–85 years and are usually asymptomatic until rupture, which is catastrophic. Aneurysm expansion can result from several factors, including direct trauma, chronic and acute infections, and inflammatory processes. Traditionally, atherosclerotic damage to the aortic wall is considered the primary risk factor for AAA formation, although this view has been recently questioned (1).

Several predisposing factors increase the likelihood of aneurysm formation, including older age, male gender, hypertension, smoking, alcohol consumption, and a family history of AAA. After age 50 in men and age 60 in women, the risk of developing an aneurysm increases with each decade (2). Men are four times more likely than women to develop an aneurysm, and having a first-degree relative with an AAA increases the risk fourfold. Smoking is the most significant modifiable risk factor, influencing both the likelihood of aneurysm development and its rate of growth.

According to the literature, more than half of patients with ruptured AAAs have previously undiagnosed aneurysms, and up to 30% of patients are initially misdiagnosed. The classic triad of symptoms—hypotension, flank or back pain, and a pulsatile mass—is reported in 25%–50% of patients with ruptured AAAs (3). Other possible manifestations include unexplained hypotension, groin pain, and sometimes pain in the lower limbs. Complications such as hematuria or gastrointestinal bleeding can also occur. Physicians must maintain a high index of suspicion and a low threshold for assessment due to the varied manifestations of this condition.

A rupture of the anterior-lateral wall of the aorta often results in immediate death, as it communicates directly with the abdominal cavity. In contrast, a rupture of the posterior-lateral wall communicates with the retroperitoneal space, providing a chance for survival. Smaller ruptures result in less blood loss and may initially seal themselves, limiting hemorrhage.

The prevalence of AAAs has decreased over time, falling from approximately 4%–8% in men over 65 to just over 2%. This decline is likely due to reduced tobacco use and healthier lifestyles. Most AAAs are small, measuring less than 5.5 cm. Rupture rates increase significantly within a year for aneurysms measuring between 5.5 and 5.9 cm, with a rate of 9.4%, making this the threshold for surgical intervention (4). An aneurysm that increases by more than 0.5 cm in size is also at high risk of rupture and requires urgent repair (5).

Although many patients are identified with AAAs (with aortic diameters exceeding 3 cm) that are suitable for preventive repair, up to 50% present with rupture as the initial symptom. The estimated mortality rate for ruptured AAAs is difficult to quantify, but historical reports indicate mortality rates of up to 90%. Modern surgical techniques have reduced this to around 75%.

The incidence of AAA rupture is 5.6–17.5 per 100,000 people per year in Western countries, with an overall patient mortality rate of approximately 80%–90% (6). AAA rupture remains a significant cause of mortality, and the aging population may lead to a further increase in cases. The only treatment option for rupture is urgent open, hybrid, or endovascular surgery.

Extracorporeal bypass is the preferred method for revascularization and limb salvage in patients with complicated AAAs and infra-renal aortic occlusion, especially when accompanied by severe comorbid conditions. This technique involves placing a graft outside the bypass arterial route and is typically used in the treatment of aorto-iliac and femoral artery diseases. Options for bypass include femoral-femoral, subclavian-femoral, axillo-femoral, obturator, thoraco-femoral, and supra-celiac or iliac-femoral bypasses (7).

Extracorporeal bypass is chosen to avoid intra-abdominal pathology and reduce the risk of transabdominal reconstruction in patients with severe visceral or systemic diseases (8). Axillo-femoral bypass is used to revascularize the lower limb when standard aorto-femoral or aorto-iliac bypass grafts cannot be used, such as in cases of infected aortic grafts (9).

In emergency departments, balloon occlusion of the aorta is often used for conditions such as traumatic intra-abdominal hemorrhage to achieve temporary hemostasis. However, placing a balloon through an AAA and its proximal neck is challenging, even under fluoroscopy, due to the aneurysm's size and the aorta's tortuosity. Therefore, aortic occlusion balloons should always be placed under fluoroscopic guidance and never blindly.

This clinical case illustrates a mycotic aneurysm complicated by an infected retroperitoneal hematoma, aligning with existing literature that highlights the high rupture risk of mycotic aneurysms even at smaller sizes (10). Despite the relatively small size of the aneurysm (3.4 × 2.9 × 2.7 cm), its mycotic nature posed a significant risk for rupture, necessitating urgent intervention. This finding is consistent with studies, such as those by Kim et al., which emphasize the life-threatening nature of mycotic aneurysms, particularly those associated with bacterial infections (10).

Mycotic aneurysms develop due to bacterial invasion of the arterial wall, a condition first described by Osler in 1885 in the context of endocarditis (11). While bacterial endocarditis remains a recognized cause, more recent studies suggest that gram-negative organisms, such as *Salmonella* and *Staphylococcus aureus*, have become the predominant pathogens, particularly in immunocompromised patients or those with pre-existing arterial disease, such as atherosclerosis (12, 13). In our case, *Escherichia coli* was isolated from the wound culture, consistent with reports of gram-negative organisms being frequent culprits in infected aneurysms. This finding underscores the importance of targeted antibiotic therapy in the management of such cases.

In this instance, the decision to perform extra-anatomical reconstruction was driven by the need to minimize the risk of reinfection in an infected surgical field. Studies support that *in situ* reconstructions using autologous veins or prosthetics carry a higher risk of graft infection, especially in the presence of active infection (14). Cryopreserved grafts and autologous vein grafts may offer lower complication rates in infected fields, although the selection of surgical technique must be tailored to the patient's condition (10).

We opted for an extraperitoneal approach to reduce the risk of peritonitis and expedite limb revascularization. The literature supports this approach in cases involving infected retroperitoneal hematomas, where avoiding contamination of the peritoneal cavity is critical (15). Additionally, the use of a balloon occlusion catheter to control intraoperative bleeding effectively minimized ischemic time and blood loss, a technique described as advantageous in cases of severe aneurysmal rupture.

Postoperatively, the patient was managed with a prolonged course of antibiotics, including meropenem and linezolid, aligning with current recommendations for treating infected aneurysms. The literature suggests that at least six weeks of antibiotic therapy is necessary to prevent recurrence of infection (16). Extended antibiotic treatment is essential for ensuring full eradication of the infection and preventing complications such as re-infection or thrombosis.

This article describes the extraperitoneal approach due to the presence of an infected retroperitoneal hematoma and the high risk of abdominal cavity infection. Diagnosis was based on clinical presentation (pain, fever), laboratory findings (inflammatory markers), and characteristic morphological features (saccular protrusion of the arterial wall, perivascular edema, hematoma, and/or fibrous tissue). Chronic contained rupture of an AAA is a rare subtype, first described by Szilagyi et al. in 1961 (16–18). It is characterized by low blood loss and stable hemodynamics due to the retroperitoneal tissue's role in sealing the rupture (19, 20).

Further examination of the patient revealed lumbar spinal tuberculosis, which likely contributed to the infra-renal AAA and its subsequent rupture. AAA rupture is typically treated with open surgery, although recent reports discuss the effectiveness of endovascular repair (21, 22). However, in cases of infected hematomas, such as this one, open extracorporeal intervention is considered optimal.

## Conclusion

Based on clinical, laboratory, and imaging data, the cause of the aneurysm and its rupture was determined to be tuberculosis spondylitis, which caused inflammation of the surrounding tissues and the aortic wall. This led to a defect in the aortic wall and subsequent bleeding. A timely diagnosis and a multidisciplinary approach allowed for thorough evaluation and selection of the optimal surgical treatment, leading to the patient's recovery.

## Data Availability

The raw data supporting the conclusions of this article will be made available by the authors, without undue reservation.
