# Supplementary material for: Complicated mycotic saccular aneurysm of the infra-renal abdominal aorta with infected retroperitoneal hematoma: a clinical case report
Source: Front Cardiovasc Med. 2024 Nov 21;11:1497561. doi: 10.3389/fcvm.2024.1497561 (PMC11617553; doi:10.3389/fcvm.2024.1497561)
Supplement: Supplementary file 1 [file Datasheet1.pdf]

Supplementary Material

Health indicators and laboratory data during hospitalization:

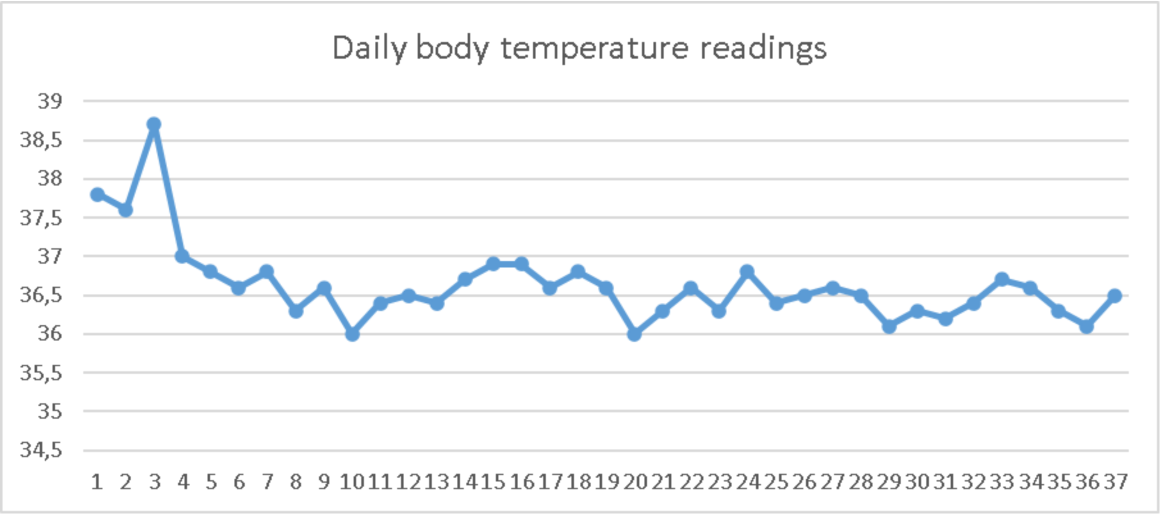

Fig 1S. In the early postoperative period, the patient experienced an increase in body temperature, which was managed with analgesics and antipyretic medications.
A subfebrile temperature persisted for a month during the postoperative period.

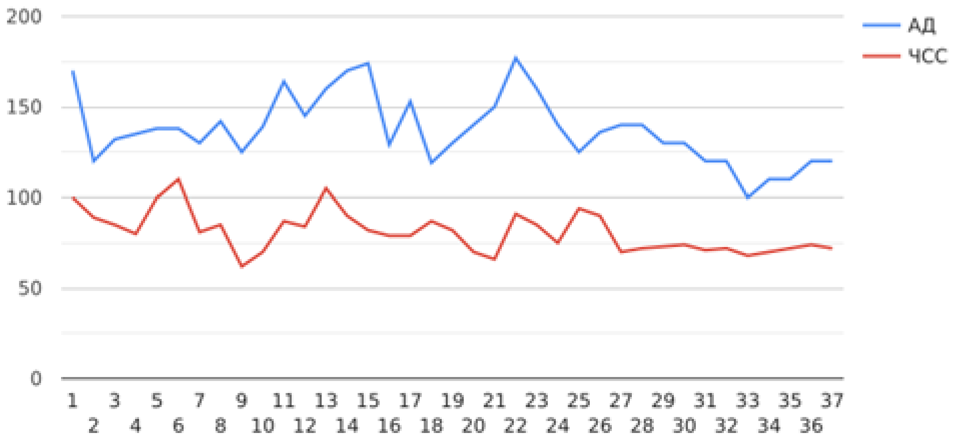

Fig 2S. During the stay in the intensive care unit, there was instability in blood pressure and heart rate. In the postoperative period, while in the specialized department, blood pressure and heart rate stabilized. In the early postoperative period, the patient exhibited signs of acute renal failure. After correction, creatinine levels normalized, and acute renal failure regressed.

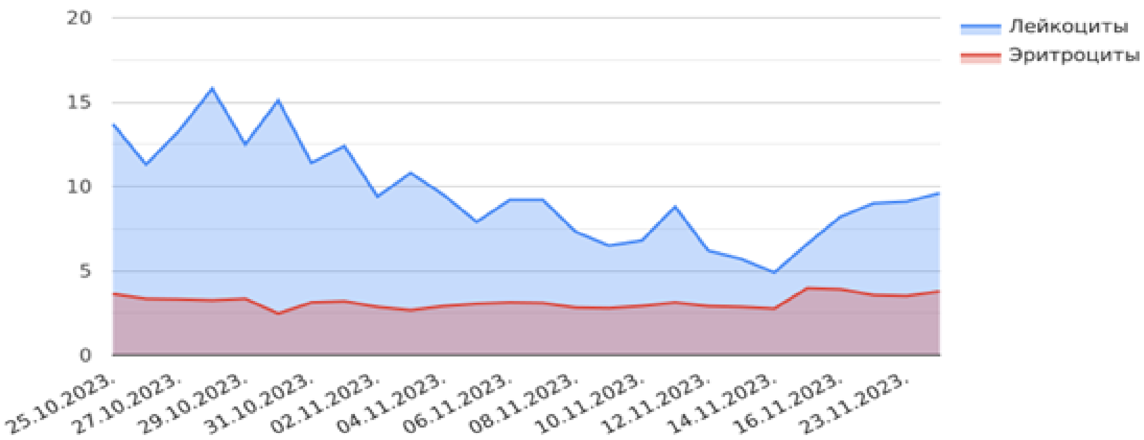

Fig 3S. Leukocyte and erythrocyte levels.

In the general blood test (GBT) – leukocytosis and increased C-reactive protein (CRP) due to the intoxication syndrome. Upon discharge, GBT, biochemical blood analysis, and CRP levels had normalized.

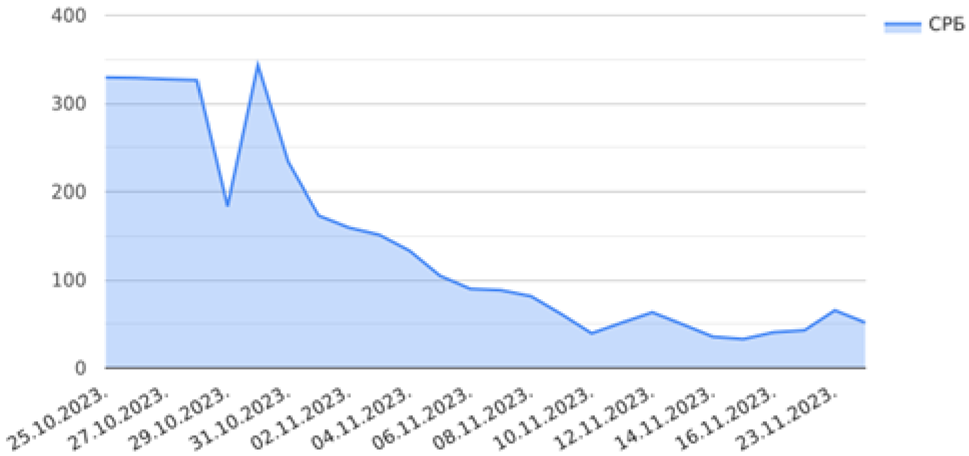

Fig 4S. Dynamics of C-reactive protein levels.

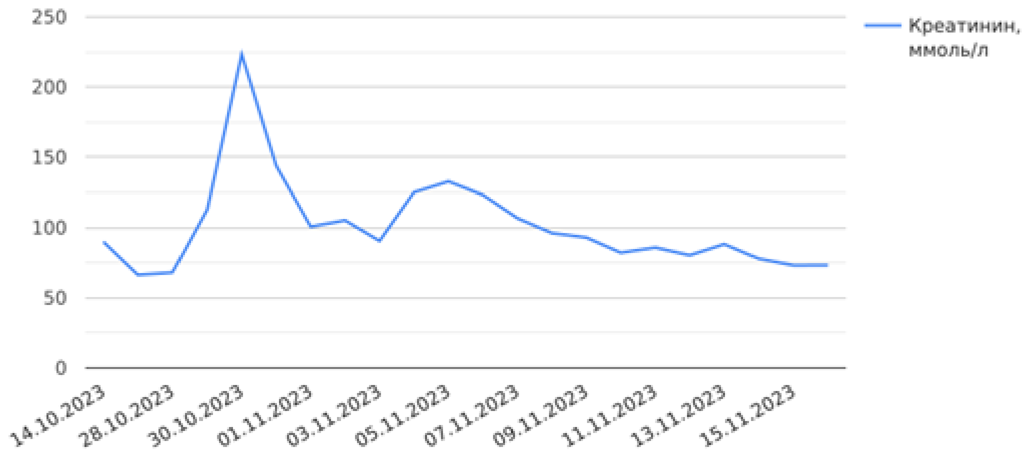

Fig 5S. Dynamics of creatinine levels.

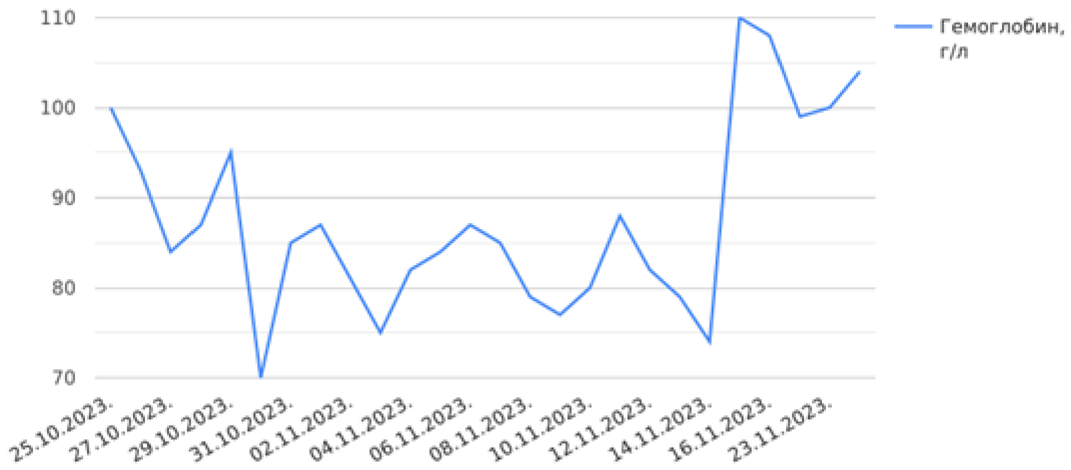

Fig 6S. Dynamics of hemoglobin levels.
